# Association of Human Papillomavirus Infection and Abnormal Anal Cytology among HIV-Infected MSM in Beijing, China

**DOI:** 10.1371/journal.pone.0035983

**Published:** 2012-04-25

**Authors:** Yu Yang, Xiangwei Li, Zhihui Zhang, Han-Zhu Qian, Yuhua Ruan, Feng Zhou, Cong Gao, Mufei Li, Qi Jin, Lei Gao

**Affiliations:** 1 MOH Key Laboratory of Systems Biology of Pathogens, Institute of Pathogen Biology, Chinese Academy of Medical Sciences & Peking Union Medical College, Beijing, China; 2 Hospital of Cancer, Chinese Academy of Medical Sciences and Peking Union Medical College, Beijing, China; 3 Vanderbilt Institute for Global Health, and Division of Epidemiology, Vanderbilt University School of Medicine, Nashville, Tennessee, United States of America; 4 State Key Laboratory for Infectious Disease Prevention and Control, National Center for AIDS/STD Control and Prevention, Chinese Center for Disease Control and Prevention, Beijing, China; University of Ottawa, Canada

## Abstract

**Background:**

In the recent years, dramatic increases in HIV transmission among men who have sex with men (MSM) have been observed in China. Human papillomavirus (HPV) infection related anal cancer is more common among HIV-infected MSM as compared to the general population. However, HPV infection and anal cytology has been rarely studied in HIV-infected MSM in China.

**Methods:**

HIV-infected MSM in Beijing, China were invited to participate in this study between January and April 2011. Anal swabs were collected for examining cytology and HPV genotypes.

**Results:**

Ninety-five eligible participants with complete questionnaire and laboratory data were included in the analyses. Thirty six of them (37.9%) showed abnormal anal cytology as follows: atypical squamous cells of undetermined significance (ASC-US) in 19 (20.0%), atypical squamous cells but cannot exclude HSIL (ASC-H) in 1 (1.1%), low-grade squamous intraepithelial lesion (LSIL) in 15 (15.8%), and high-grade squamous intraepithelial lesion (HSIL) in 1 (1.1%). HPV6 (20.0%), HPV16 (10.9%), HPV56 (10.9%), HPV52 (9.1%) and HPV39 (9.1%) were observed most frequently among those with normal anal cytology, while different distribution was found in the ones with abnormal anal cytology as HPV6 (19.4%), HPV16 (19.4%), HPV45 (16.7%), HPV52 (16.7%) and HPV18 (11.1%). In addition, HPV16, HPV45, HPV52 and HPV18 were the most frequent high-risk types in patients with abnormal anal cytology. HPV multiplicity was found to be significantly related to the prevalence of abnormal anal cytology (p for trend = 0.04).

**Conclusions:**

High prevalence of HPV infection and abnormal anal cytology was observed among HIV-infected MSM in China. Infection of multiple HPV types or high-risk types was found to be associated with an increased risk of abnormal anal cytology.

## Introduction

While anal carcinoma is rare in the general population [1], it is more common among men who have sex with men (MSM) [2], [3] and individuals with human immunodeficiency virus (HIV) infection [4], [5]. In the past decades, the incidence of anal cancer has been increasing in both general MSM and HIV-infected MSM [6], [7]. Screening of anal precancerous lesions in HIV-infected MSM has been suggested to be important for cancer prevention [8]. In China, dramatic increases in HIV transmission among MSM have been observed in recent years. MSM accounted for nearly one-third of new HIV infections in 2009 [9]. Anal screening should be attached importance to this high-risk population. Unfortunately, high-resolution anoscopy is still a resource that is both costly and difficultly to access for HIV-infected MSM in China at present. Anal cytology, despite of its high sensitivity and low specificity characteristics, should be much helpful to this target population for monitoring the development of anal precancerous lesions [10], [11].

Human papillomavirus (HPV) infection, especially high-risk HPV (HR-HPV) viral types such as HPV 16 and HPV 18, is a significant risk factor for development of anal intraepithelial neoplasia (AIN) and anal cancer [12], [13]. Two recent meta-analyses have shown that 73.4% and 65.6% of all anal cancer are caused by HPV 16 [14], [15]. HPV has not only been linked to the transformation from innocent cervical epithelium into dysplastic precancerous states known as cervical interstitial neoplasia (CIN), but has also been associated with the development of anal squamous intraepithelial lesions (ASIL) [16]. Other risk factors include: receptive anal intercourse, history of sexually transmitted disease, number of lifetime sexual partners, HIV status, lower CD4 cell count, immunosuppression after solid-organ transplantation, and current cigarette smoking [17], [18]. Recent studies have demonstrated that more than 90% of HIV positive MSM were also infected with HPV [18]–[21]. This particular group has a very high risk of developing ASIL and anal cancer [18]. HPV genotyping and anal cytology are important to assess the risks of HPV-related anal diseases in high-risk populations. To our knowledge, this topic has not yet been studied in China. The objective of this pilot cross-sectional study was to investigate the prevalence and distribution of HPV genotypes in HIV-infected MSM with abnormal anal cytology in Beijing, China.

## Results

### Subject recruitment and characteristics

A total of 98 eligible participants gave consent to be included into the study. Three of them were exclude in the final data analysis because of unsatisfactory cytological evaluation. Of the remaining participants, about three quarters (75.1%) were aged 20–39 years (Table 1). The majority was of Han ethnic (94.7%), and over half had been educated over 12 years (56.8%). Around 72.6% described themselves as homosexuals, and 85.3% of these had their first homosexual intercourse at 18 years old or later. More than half (53.7%) reported a history of sexually transmitted diseases (STD) other than HIV, and 54.7% were within the first 2 years of their HIV diagnosis. Over sixty (65.3%) of participants had a recent CD4 cell count between 200 to 500 cells/µL of blood, and 30.5% participants ever had highly active antiretroviral therapy (HAART) (Table 1). Forty-nine participants (51.6%) got positive results in syphilis test. Only three subjects showed abnormal physical signs related to STD and two of them were newly diagnosed with anal warts (2.1%).

**Table 1:** Major characteristics of the study participants.

Variables	n*	%
**Total**	95	100
**Age**		
≤19 years	3	3.2
20–29 years	33	34.7
30–39 years	38	40.4
≥40 years	21	22.1
**Ethnicity**		
Han	90	94.7
Others	5	5.3
**Education**		
≤9 years	16	16.8
10–12 years	25	26.3
>12 years	54	56.8
**Marriage status**		
Unmarried	57	60.0
Married/divorced/widowed	38	40.0
**Self-reported sexual orientation**		
Homosexual	69	72.6
Bisexual	25	26.3
**History of sexual activity with female**		
No	43	45.3
Yes	52	54.7
**History of STD (except HIV)**		
No	40	42.1
Yes	51	53.7
**Time after HIV diagnosis**		
<2 year	52	54.7
2–3year	27	28.4
>3 years	16	16.8
**History of HARRT**		
No	64	67.4
Yes	29	30.5
**Latest CD4 cell count (cells/µL of blood)** #		
<200	7	7.4
200–500	62	65.3
>500	14	14.7
**Age at the first homosexual act**		
<18	14	14.7
≥18	81	85.3

Abbreviation: HAART = highly active antiretroviral therapy; HIV = human immunodeficiency virus; STD = sexually transmitted diseases.

* Sum may not always add up to total because of missing data.

# CD4 cell count was measured in the past three months.

### Association between sociodemographic/sexual factors and abnormal anal cytology

As shown in Table 2, of the study participants, 37.9% (36/95) had abnormal anal cytology, 20.0% (19/95) had atypical squamous cells of undetermined significance (ASC-US), 1.1% (1/95) with atypical squamous cells but cannot exclude HSIL (ASC-H), 15.8% (15/95) with low-grade squamous intraepithelial lesion (LSIL), and 1.1% (1/95) with high-grade squamous intraepithelial lesion (HSIL). Half of the participants who had been educated equal to or less than nine years showed abnormal anal cytology. As the CD4 count (cells/µL of blood) increases, study participants appear more likely to have normal anal cytology (<200: 42.9%; 200–500: 54.8%; >500: 78.6%). Among participants with CD4 count <200, 57.1% had abnormal anal cytology and 42.9% had ASIL/ASC-H. Among participants who had ever received HAART, 48.3% had abnormal anal cytology while only 17.2% had ASIL/ASC-H. Among participants who had been diagnosed as HIV positive for less than 2 years, 40.4% had abnormal anal cytology and 23.1% had ASIL/ASC-H. On the other hand, among participants who had been diagnosed as HIV positive for more than 3 years, 31.3% had abnormal anal cytology and no one had ASIL/ASC-H. A higher prevalence of abnormal anal cytology was observed among those taking receptive anal sex as a regular homosexual behavior (Table 3 and Table 4). In addition, a positive history of STD was found to be related with an increased risk of ASIL/ASC-H (12/19 vs. 4/16, p = 0.01) among those with abnormal anal cytology.

**Table 2:** Association between sociodemographics and abnormal anal cytology among study participants.

Variables	Normal n/N* (%)	Abnormal		
		Total n/N* (%)	ASC-US n/N* (%)	ASIL/ASC-H n/N* (%)
**Total**	59/95 (62.1)	36/95 (37.9)	19/95 (20.0)	17/95 (17.9)
**Age**				
<20 years	3/3 (100.0)	0/3 (0)	0/3 (0)	0/3 (0)
20–29 years	20/33 (60.6)	13/33 (39.4)	6/33 (18.2)	7/33 (21.2)
30–39 years	20/38 (52.6)	18/38 (47.4)	11/38 (28.9)	7/38 (18.4)
>39 years	16/21 (76.2)	5/21 (23.8)	2/21 (9.5)	3/21 (14.3)
p for difference#		0.20	0.30	0.94
**Ethnicity**				
Han	56/90 (62.2)	34/90(37.8)	18/90 (20.0)	16/90 (17.8)
Others	3/5 (60.0)	2/5 (40.0)	1/5 (20.0)	1/5 (20.0)
p for difference#		1.00	1.00	1.00
**Education**				
≤9 years	8/16 (50.0)	8/16 (50.0)	5/16 (31.3)	3/16 (18.8)
10–12 years	16/25 (64.0)	9/25 (36.0)	4/25 (16.0)	5/25 (20.0)
>12 years	35/54 (64.8)	19/54 (35.2)	10/54 (18.5)	9/54 (16.7)
p for difference#		0.55	0.45	0.93
**Marriage status**				
Unmarried	39/57 (68.4)	18/57 (31.6)	9/57 (15.8)	9/57(15.8)
Married/divorced/widowed	20/38 (52.6)	18/38 (47.4)	10/38 (26.3)	8/38 (21.1)
p for difference#		0.12	0.21	0.51
**History of HARRT**				
No	43/64 (67.2)	21/64 (32.8)	10/64 (15.6)	11/64 (17.2)
Yes	15/29 (51.7)	14/29 (48.3)	9/29 (31.0)	5/29 (17.2)
p for difference#		0.15	0.09	1.00
**Latest CD4 cell count (cells/µL of blood)** §				
<200	3/7 (42.9)	4/7 (57.1)	1/7 (14.3)	3/7 (42.9)
200–500	34/62 (54.8)	28/62 (45.2)	16/62 (25.8)	12/62(19.4)
>500	11/14 (78.6)	3/14 (21.4)	2/14 (14.3)	1/14 (7.1)
p for difference#		0.21	0.60	0.16
**Time since HIV diagnosis (years)**				
<2	31/52 (59.6)	21/52 (40.4)	9/52 (17.3)	12/52 (23.1)
2–3	17/27 (63.0)	10/27 (37.0)	5/27 (18.5)	5/27 (18.5)
>3	11/16 (68.8)	5/16 (31.3)	5/16 (31.3)	0/16 (0)
p for difference#		0.80	0.46	0.09

Abbreviation: ASCUS = atypical squamous cells of undetermined significance; ASC-H = atypical squamous cells but cannot exclude HSIL; ASIL = anal squamous intraepithelial lesions; HAART = highly active antiretroviral therapy; HIV = human immunodeficiency virus.

* Sum may not always add up to total because of missing data.

# Participants with normal cytology was treated as control.

§ CD4 cell count was measured in the past three months.

**Table 3:** Association between sexual factors and abnormal anal cytology among study participants.

Variables	Normal n/N* (%)	Abnormal		
		Total n/N* (%)	ASCUS n/N* (%)	ASIL/ASC-H n/N* (%)
**History of STD (except for HIV)**				
No	24/40 (60.0)	16/40 (40.0)	12/40 (30.0)	4/40 (10.0)
Yes	32/51 (62.7)	19/51 (37.3)	7/51 (13.7)	12/51 (23.5)
p for difference		0.79	0.06	0.09
**Number of homosexual partners ever had**				
<10	21/34 (61.8)	13/34 (38.2)	7/34 (20.6)	6/34 (17.6)
10–50	22/40 (55.0)	18/40 (45.0)	9/40 (22.5)	9/40 (22.5)
>50	12/17 (70.6)	5/17 (29.4)	3/17 (17.6)	2/17 (11.8)
p for difference		0.53	0.92	0.62
**Age at the first homosexual act**				
<18	11/14 (78.6)	3/14 (21.4)	2/14 (14.3)	1/14 (7.14)
≥18	48/81 (59.3)	33/81 (40.7)	17/81 (21.0)	16/81 (19.8)
p for difference		0.17	0.73	0.45
**Is anal sex a regular homosexual behavior?**				
No	8/11 (72.7))	3/11 (27.3)	0/11 (0)	3/11 (27.3)
Yes	51/84 (60.7)	33/84 (39.3)	19/84 (22.6)	14/84 (16.7)
p for difference		0.53	0.11	0.41
**Is receptive anal sex a regular homosexual behavior?**				
No	37/53 (69.8)	16/53 (30.2)	7/53 (13.2)	9/53 (17.0)
Yes	22/42 (52.4)	20/42 (47.6)	12/42 (28.6)	8/42 (19.0)
p for difference		0.08	0.06	0.79
**Is insertive anal sex a regular homosexual behavior?**				
No	30/52 (57.7)	22/52 (42.3)	12/52 (23.1)	10/52 (19.2)
Yes	29/43 (67.4)	14/43 (32.6)	7/43 (16.3)	7/43 (16.3)
p for difference		0.33	0.41	0.71
**Is oral sex a regular homosexual behavior?**				
No	3/6 (50.0)	3/6 (50.0)	1/6 (16.7)	2/6 (33.3)
Yes	56/89 (62.9)	33/89 (37.1)	18/89 (20.2)	15/89 (16.9)
p for difference		0.67	1.00	0.29
**Is anilinction a regular homosexual behavior?**				
No	44/68 (64.7)	24/68 (35.3)	12/68 (17.6)	12/68 (17.6)
Yes	15/27 (55.6)	12/27 (44.4)	7/27 (25.9)	5/27 (18.5)
p for difference		0.41	0.36	1.00
**Frequency of homosexual behaviors in the past 6 months**				
<once a week	48/77 (62.3)	29/77 (37.7)	15/77 (19.5)	14/77 (18.2)
≥once a week	10/17 (58.8)	7/17 (41.2)	4/17 (23.5)	3/17 (17.6)
p for difference		0.79	0.74	1.00

Abbreviation: ASCUS = atypical squamous cells of undetermined significance; ASC-H = atypical squamous cells but cannot exclude HSIL; ASIL = anal squamous intraepithelial lesions, include low-grade squamous intraepithelial lesion (LSIL) and high-grade squamous intraepithelial lesion (HSIL); HIV = human immunodeficiency virus; STD = sexually transmitted diseases.

* Sum may not always add up to total because of missing data.

**Table 4:** Association between sexual factors and abnormal anal cytology among study participants.

Variables	Normal n/N * (%)	Abnormal		
		Total n/N* (%)	ASCUS n/N* (%)	ASIL/ASC-H n/N* (%)
**Number of partners with oral sex in the past 6 months**				
≤2	22/38 (57.9)	16/38 (42.1)	6/38 (15.8)	10/38 (26.3)
>2	13/18 (72.2)	5/18 (27.8)	5/18 (27.8)	0/18 (0)
p for difference		0.30	0.31	0.02
**Condom use during oral sex in the past 6 months**				
Always	6/11 (54.5)	5/11 (45.5)	2/11 (18.2)	3/11 (27.3)
Sometimes/Never	29/45 (64.4)	16/45 (35.6)	9/45 (20.0)	7/45 (15.6)
p for difference		0.73	1.00	0.40
**Number of partners with insertive sex in the past 6 months**				
≤2	19/31 (61.3)	12/31 (38.7)	8/31 (25.8)	4/31 (12.9)
>2	11/14 (78.6)	3/14 (21.4)	2/14 (14.3)	1/14 (7.1)
p for difference		0.32	0.47	1.00
**Condom use during insertive anal sex in the past 6 months**				
Always	27/39 (69.2)	12/39 (30.8)	8/39 (20.5)	4/39 (10.3)
Sometimes/Never	5/7 (71.4)	2/7 (28.6)	1/7 (14.3)	1/7 (14.3)
p for difference		1.00	1.00	1.00
**Number of partners with receptive anal sex in the past 6 months**				
≤2	19/34 (55.9)	15/34 (44.1)	6/34 (17.6)	9/34 (26.5)
>2	14/18 (77.8)	4/18 (22.2)	4/18 (22.2)	0/18 (0)
p for difference		0.12	0.73	0.02
**Condom use during receptive anal sex in the past 6 months**				
Always	28/46 (60.9)	18/46 (39.1)	10/46 (21.7)	8/46 (17.4)
Sometimes/Never	7/8 (87.5)	1/8 (12.5)	0/8 (0)	1/8 (12.5)
p for difference		0.24	0.33	1.00
**Have received anilinction in the past 6 months**				
No	54/84 (64.3)	30/84 (35.7)	15/84 (17.9)	15/84 (17.9)
Yes	5/11 (45.5)	6/11 (54.5)	4/11 (36.4)	2/11 (18.2)
p for difference		0.32	0.22	1.00
**Syphilis infection** #				
Negative	31/49 (63.3)	18/49 (36.7)	8/49 (16.3)	10/49 (20.4)
Positive	28/46 (60.9)	18/46 (39.1)	11/46 (23.9)	7/46 (15.2)
p for difference		0.81	0.36	0.51

Abbreviation: ASCUS = atypical squamous cells of undetermined significance; ASC-H = atypical squamous cells but cannot exclude HSIL; ASIL = anal squamous intraepithelial lesions, include low-grade squamous intraepithelial lesion (LSIL) and high-grade squamous intraepithelial lesion (HSIL).

* Sum may not always add up to total because of missing data.

# Syphilis positivity was determined through screening by a positive rapid-plasma reagin test and confirmation by an Enzyme-linked immunosorbent assay for detection of antibodies to Treponema pallidum.

### HPV genotypes and abnormal anal cytology

Table 5 shows the distribution of HPV genotypes of the study participants. Among those with abnormal anal cytology (n = 36), 77.8% were positive for at least one of the 26 targeted HPV types, and 44.4% were infected with multiple HPV type while this prevalence was only 20% in those with normal anal cytology. Among 17 participants with ASIL/ASC-H, 87.5% were infected with at least one type of HPV. The most common types among those with normal anal cytology were HPV6 (20.0%), HPV16 (10.9%), HPV56 (10.9%), HPV52 (9.1%) and HPV39 (9.1%). For those with abnormal anal cytology, the most prevalent types were HPV6 (19.4%), HPV16 (19.4%), HPV45 (16.7%), HPV52 (16.7%) and HPV18 (11.1%) were observed most frequently.

**Table 5:** HPV genotypes and abnormal anal cytology among study participants.

HPV infection	Normal (N* = 55) n (%)	Abnormal		
		Total (N = 36) n (%)	ASCUS (N = 19) n (%)	ASIL/ASC-H (N = 17) n (%)
***Total***				
Any type	36 (65.5)	28 (77.8)	14 (73.7)	14 (82.4)
Single type	25 (45.5)	12 (33.3)	6 (31.6)	6 (35.3)
Multiple type	11 (20.0)	16 (44.4)	8 (42.1)	8 (47.1)
***High-risk types***				
HPV16	6 (10.9)	7 (19.4)	5 (26.3)	2 (11.8)
HPV18	3 (5.5)	4 (11.1)	1 (5.3)	3 (17.6)
HPV31	1 (1.8)	1 (2.8)	1 (5.3)	0 (0)
HPV33	1 (1.8)	0 (0)	0 (0)	0 (0)
HPV35	0 (0)	0 (0)	0 (0)	0 (0)
HPV39	5 (9.1)	3 (8.3)	1 (5.3)	2 (11.8)
HPV45	2 (3.6)	6 (16.7)	2 (10.5)	4 (23.5)
HPV51	0 (0)	0 (0)	0 (0)	0 (0)
HPV52	5 (9.1)	6 (16.7)	2 (10.5)	4 (23.5)
HPV56	6 (10.9)	3 (8.3)	1 (5.3)	2 (11.8)
HPV58	0 (0)	1 (2.8)	0 (0)	1 (5.9)
HPV59	1 (1.8)	2 (5.6)	2 (10.5)	0 (0)
HPV66	2 (3.6)	0 (0)	0 (0)	0 (0)
HPV68	4 (7.3)	3 (8.3)	1 (5.3)	2 (11.8)
HPV82	2 (3.6)	3 (8.3)	2 (10.5)	1 (5.9)
HPV83	0 (0)	0 (0)	0 (0)	0 (0)
HPV53	0 (0)	0 (0)	0 (0)	0 (0)
HPV26/55	1 (1.8)	2 (5.6)	2 (10.5)	0 (0)
***Low-risk types***				
HPV06	11 (20.0)	7 (19.4)	3 (15.8)	4 (23.5)
HPV11	3 (5.5)	3 (8.3)	1 (5.3)	2 (11.8)
HPV40/42/44	1 (1.8)	0 (0)	0 (0)	0 (0)
HPV61/73	0 (0)	1 (2.8)	1 (5.3)	0 (0)

Abbreviation: ASCUS = atypical squamous cells of undetermined significance; ASC-H = atypical squamous cells but cannot exclude HSIL; ASIL = anal squamous intraepithelial lesions, include low-grade squamous intraepithelial lesion (LSIL) and high-grade squamous intraepithelial lesion (HSIL); HPV = human papillomavirus.

* Four study participants were not included in this analysis due to missing data on HPV genotypes.

### The association between anal HR-HPV infection and anal cytology result

As shown in Table 6, having a high-risk HPV type infection was at an increased risk of ASIL/ASC-H with an adjusted odds ratio (OR) of 2.48 (95% confidence interval (95% CI): 0.68–9.00). Having a high-risk HPV type infection is associated with a 63% increased risk of ASC-US (adjusted OR: 1.63; 95% CI: 0.49–5.43). There was a significant association between HPV multiplicity and the prevalence of abnormal anal cytology. Increased risks were observed for subjects carrying more HPV types (p for trend = 0.04). Individuals positive for three or more HPV types were at a significantly increased risk of abnormal anal cytology with an adjusted OR of 4.71 (95% CI: 1.02–21.76) compared to those HPV anal infection negative, these individuals were also at a high risk of ASIL/ASC-H with an adjusted OR of 7.49 (1.07–52.21).

**Table 6:** Association of abnormal anal cytology and HPV genotypes among study participants.

Anal infection of 26 HPV types	Abnormal/Normal	Adjusted OR* (95% CI)	ASCUS/Normal	Adjusted OR* (95% CI)	(ASIL/ASC-H)/Normal	Adjusted OR* (95% CI)
**High-Risk types infection**						
Negative for high-risk types	10/26	Ref.	6/26	Ref.	4/26	Ref.
Positive for high-risk types	26/29	2.02 (0.77–5.29)	13/29	1.63 (0.49–5.43)	13/29	2.48 (0.68–9.00)
p for difference		0.15		0.43		0.17
**By multiplicity of HPV types**						
0	8/19	Ref.	5/19	Ref.	3/19	Ref.
1	12/25	1.22 (0.40–3.72)	6/25	0.83 (0.20–3.41)	6/25	1.71 (0.36–8.02)
2	9/7	3.81 (1.00–14.61)	5/7	4.08 (0.79–21.11)	4/7	4.71 (0.77–28.61)
≥3	7/4	4.71 (1.02–21.76)	3/4	2.71(0.41–17.82)	4/4	7.49 (1.07–52.21)
p for trend		0.04		0.20		0.04

Abbreviation: ASCUS = atypical squamous cells of undetermined significance; ASC-H = atypical squamous cells but cannot exclude HSIL; ASIL = anal squamous intraepithelial lesions, include low-grade squamous intraepithelial lesion (LSIL) and high-grade squamous intraepithelial lesion (HSIL); CI = confidence intervals; HPV = human papillomavirus; OR = odds ratio.

* Adjusted for age, education, age at the first homosexual act, frequency of homosexual behaviors in the past 6 months and ever found sexual partners in gay venues.

## Discussion

To our knowledge, this pilot study was the first investigation into the prevalence of HPV genotypes in HIV-infected MSM with abnormal anal cytology in China. Based on a cross-sectional study design, a total of 95 HIV-infected MSM were included in the study, 36 of them (37.9%) showed abnormal anal cytology including 17 with ASIL/ASC-H (17.9%). Different HPV genotype distributions were found between patients with normal and abnormal anal cytology. Infection of multiple HPV types and high-risk types were found to be associated with an increased risk of abnormal anal cytology.

Invasive anal cancer has well-documented precursors, known as HSIL (cytology) or AIN2/3 (histology) [22]. LSIL and AIN 1 are not considered to be direct precursors of invasive anal cancer, but may precede the later development of HSIL or AIN2/3. Therefore, ASIL, which include LSIL and HSIL, was considered as AIN to predict the presence of anal dysplasia. Palefsky JM and colleagues reported the prevalence of AIN2/3 as 52% in 81 HIV-positive MSM participants from US in 2005 [23]. In 2010, Salit IE and colleges assessed the prevalence of abnormal anal cytology based on a cross-sectional study of 401 HIV-positive MSM from Canada. They found cytology was abnormal in 67% of patients: HSIL, 12%; LSIL, 43% and ASC-US, 12% [24]. In addition, when de Pokomandy A and colleagues performed anal cytology and high-resolution anoscopy (HRA) in the baseline screening of a cohort study with 247 HIV- seropositive MSM, the anal abnormal cytology results were ASCUS, 33%; LSIL, 26%; HSIL, 6%, while HRA findings were AIN1, 50%; AIN2, 17% AIN3, 13% [25]. In Asians, Li AH and colleagues reported the prevalence of anal abnormal cytology among HIV positive MSM in in Thailand: 16.1% had ASC-US, 14.4% had LSIL and 3.4% had HSIL [26]. In our present study, the prevalence of abnormal anal cytology was 37.9%: ASC-US, 20.0%; LSIL, 15.8%; HSIL, 1.1%. Our results were very close to the data from Thailand but not the data from US and Canada especially with respect to the prevalence of HSIL. While it is difficult to directly compare the results from different studies due to the various characteristics of the study participants including age, stage of disease, status of immune system, and history of HAART. As reported, current CD4 cell count, CD4 cell count at beginning of HAART and duration of HAART might influence risks of AIN2/3 among HIV-infected MSM [25]. Consistently, our results also observed increased prevalence of normal anal cytology among those with higher current CD4 cell count (p for trend = 0.04). Despite of the heterogeneity between studies, it is consistent that abnormal anal cytology is common in MSM with HIV infection. Considering anal cancer risk in this population is present and increasing, anal screening could prove to be important [27]. Although anal cytology testing is currently imperfect with low specificity, it is still a useful tool in identifying high-risk patients with HIV infection in regions with limited clinical resources. In addition, it also provides an important support for the study addressing the impact of specific HPV types during the development of anal cancer.

In the past decade, multiple studies have reported that anal HPV infection is common among MSM especially in HIV-infected MSM [6], [28]–[30]. In our previous study, the prevalence of HPV infection was reported as 96% in newly diagnosed HIV-infected Chinese MSM [19]. In the present study the prevalence of HPV infection was 70.3%. Considering 30.5% of study participants were receiving HAART and only 7.4% of them with low CD4 cell counts less than 200 cells/µL of blood, the negative correlation between anal HPV infection and CD4 cell count might be a possible explanation to the observed lower HPV prevalence [31]. In addition, although it has been consistently reported that HAART had little effect on ASIL and anal cancer in HIV-infected MSM [32], [33], the role of HAART on the natural history of anal HPV infection including clearance and acquisition is still not clear [20], [32]. Studies with larger sample sizes are needed to address this important issue and to explore ways of controlling HPV infection and related disease in patients with HIV infection. Consistent with previous reports, multiple HPV type infection was common in our study participants, HPV multiplicity and high-risk HPV types were associated with increased risks of ASIL. Among those with abnormal anal cytology, the most common high-risk types were found to be HPV16 (19.4%), HPV45 (16.7%), HPV52 (16.7%) and HPV18 (11.1%) [32]. In a recently published article from Germany, it was reported that the most common high-risk HPV types were HPV16 (72.5%), HPV18 (25%) and HPV68 (12.5%) in HIV-infected MSM with HSIL [34]. The different prevalence and distribution of HPV subtypes in different study populations should be given exclusive attention, because it might reflect various geographic distribution of the genotypes or heterogeneous host susceptibility to the pathogens. This important issue is also crucial for the development of specific prevention strategies including vaccine. In addition, the different prevalence of HPV types might contribute to, at least in part, the lower prevalence of HSIL (1.1%) observed in our study as well. Further studies with large sample size are needed to verify these important postulations.

Some limitations of this study should be considered. First, HRA needs more advanced medical recourses especially for HIV-infected patients [8], to our knowledge, no hospital provides HRA for HIV infections in China currently. As recently reported [35], in HIV-infected MSM, the direct use of HRA is the most cost-effective strategy for detecting AIN 2/3. However, the higher cost per use for HRA was offset by the high sensitivity and low specificity of HPV and cytology testing. Therefore, the major aim of this pilot study focused on the investigation of the association between HPV prevalence and abnormal anal cytology. At the present time, we are trying to set up HRA for HIV-infected MSM under cooperation with infectious diseases hospital which would be much helpful for anal cancer screening among HIV-infected MSM. Second, with respect to the observed extremely lower prevalence of HSIL (1.1%) as compare to the previous reports (6%–12%) [24], [25], the potential misclassification of anal cytology in our study could not be completely excluded. The techniques of cervical cytology has been mature in China, however, training for anal cytologist still should be strengthened in China. Third, socio-demographic data and risk behaviors were based on an interview using a standardized questionnaire which is open to recall and information bias. Forth, the small sample size of this pilot study limited further analysis, and potential selection bias cause by the used sampling method should be kept in mind as well when interpreting the prevalence data.

In conclusion, the high prevalence of HPV infection and abnormal anal cytology was observed among HIV-infected MSM in China. Infection of multiple HPV types and high-risk types were found to be associated with an increased risk of abnormal anal cytology. Considering the high prevalence of HIV infection in this high-risk population in China, more studies addressing anal HPV infection and related diseases in HIV-infected MSM are needed.

## Materials and Methods

### Ethic review

The study was approved by the Ethics Committee of the Institute of Pathogen Biology, Chinese Academy of Medical Sciences & Peking Union Medical College. Written informed consent was obtained from each study participant before the interview, sample collection and testing.

### Study population

Study participants were outpatients of the Capital City Dermatology Hospital and were recruited through local MSM nongovernment organization (Beijing Rainbow Volunteers Workstation) between January and April 2011. To maximize outreach of study information to HIV-infected MSM community, multiple methods were used for recruitment including MSM website advertisement, distributing flyers with study-related information at MSM-frequented venues (e.g., MSM clubs, bars, parks and bathhouses), and participants were also encouraged to refer their friends and acquaintances to attend the study. Those eligible to participate were males, 18 years or older, HIV positive, ever had homosexual behaviors, willing to take tests for anal HPV infection and anal cytology, and provision of written informed consent. The presence or absence of any symptoms, including anal symptoms, did not influence eligibility. Study participants who were positive for suspicious symptoms related to STD (purulent secretion, rash, ulcer, pain, abnormal outgrowth and pruritus on the penis or crissum) and abnormal cytology were informed by study personnel in private and referred to the Capital City Dermatology Hospital or the Beijing Cancer Hospital for further diagnosis and treatment.

### Data collection

Sociodemographic and sexual behavior data were collected using one-to-one interviews in a separate room and a standardized questionnaire. The content of the questionnaire includes self-reported sociodemographic characteristics (e.g., age, income, ethnicity, education, employment, and marriage status), regular homosexual behavior, sexual behaviors in the past 6 months, status of HIV infection and treatment, knowledge on STD, history of STD, and lifestyle factors (i.e. current drinking status, history of drug abuse and injection drug use). As explained to the participants, regular homosexual behavior was defined as at least 50% of sexual activities occurring with males. Each study participant was assigned a unique code that was used to link the questionnaire and specimens. Personal contact information, which was blinded to researchers, was kept by the Beijing Rainbow Volunteers Workstation for test results feedback and data validation.

### Cytological analysis and HPV DNA typing

Trained personnel collected anal samples for cytological analysis and HPV typing by rotating a saline water moistened nylon flocked swab in the anal canal for about 2 minutes. The cytological specimens were then placed on a glass slide and stained with a Papanicolaou's stain. The cytology slides were read by a cytologist from Hospital of Cancer, Chinese Academy of Medical Sciences and Peking Union Medical College (Dr. Zhihui Zhang). Using the Bethesda system criteria for evaluation of cervical cytological results [36], the anal cytological results were classified as normal and abnormal. Abnormal results were further classified as ASC-US, ASC-H, LSIL and HSIL [37]. Smears were classified as unsatisfactory if no epithelial cells were present. After cytological samples have been collected, the swab was then kept in a 3 mL sample transport medium of the Tellgenplex™ HPV DNA Test (Tellgen Life Science, Shanghai, China). Tellgenplex™ HPV DNA Test is based on suspension bead array method to identify HPV types. Suspension bead array is a technical based on PCR, beads coated hybridization, flow cytometry, lasers and digital signal processing. Target HPV DNA-specific probes can be coated on the surface of spectrally addressable polystyrene beads. As the beads are internally labeled with a ratio of two spectrally distinct fluorophores, the bead lots are assignable to class-specific HPV subtypes. Mixtures of different bead suspensions can be used in the same well of a 96-well plate format allowing for multiplex analysis. Tellgenplex™ HPV DNA Test provides identification of 19 recommended high-risk subtypes (16, 18, 26, 31, 33, 35, 39, 45, 51, 52, 53, 55, 56, 58, 59, 66, 68, 82, and 83) which are associated with cervical cancer. Besides that, the kit also provides detection of 7 low-risk subtypes (6, 11, 40, 42, 44, 61, and 73). The tests were performed by Xiangwei Li and Yu Yang in the laboratory located in the institute of Pathogen Biology as described in elsewhere [19].

### Statistical analysis

Double entered questionnaires and laboratory results were analyzed using Statistical Analysis System (SAS 9.2 for Windows; SAS Institute Inc., NC, USA). The study population was described by age, ethnicity, education, marriage status, age at the first homosexual act, number of homosexual partners ever had, status of syphilis, ever had STD other than HIV, had receipted HAART, latest CD4 cell count (tested in the recent three months), time since HIV diagnosis, regular sex behavior (anal sex, receptive anal sex, insertive anal sex, oral sex, anilinction), number of partners in the past six months and condom use during these activities. Differences between groups were assessed using Pearson chi-square or Fisher exact test.

The associations of potential risk factors with HPV infection and anal cytological results were estimated by means of OR and 95% confidence intervals (CI) using logistic regression analysis. Covariates with a significant association in the univariate analyses were included the following multivariate analysis using a significance level of 0.05. In order to investigate the combined effect of HPV types, the risk of anal cytological results was then assessed with respect to the HPV subgroups according to carcinogenicity and the number of HPV types in each sample. Tests for trend by were performed by treating categories of the number as continuous variable in the logistic regression analysis.
